# Carer perceptions of inpatient psychiatric hospitals and the treatment of their loved ones: A meta-review of the literature

**DOI:** 10.1177/00048674261478446

**Published:** 2026-08-18

**Authors:** Katarina Khitra, Matthew Modini, Maree J Abbott

**Affiliations:** 1 School of Psychology, The University of Sydney, Camperdown, NSW, Australia; 2 Concord Centre for Mental Health, Sydney Local Health District, NSW Health, Australia

**Keywords:** carer, caregiver, perception, psychiatric hospital, mental illness, systematic review, meta-review

## Abstract

**Background:**

Inpatient psychiatric admission remains a key intervention in severe mental illness; however, less is known about how informal carers perceive these settings. A meta-review, that is, a systematic review of systematic reviews, was conducted to synthesise factors shaping carers’ perceptions of inpatient psychiatric hospitals.

**Method:**

MEDLINE, PsycINFO and EMBASE were systematically searched for eligible reviews. Methodological quality was appraised using the AMSTAR-2 framework adapted for qualitative evidence, excluding reviews rated “Critically Low.” Thematic synthesis was used to detect key overarching factors that generalised across multiple reviews.

**Results:**

Seventeen systematic reviews were included, from which five key themes were identified: exclusion and disempowerment; communication and information deficits; emotional and care burden; systemic barriers and crisis-driven pathways and a desire for partnership and collaborative care.

**Conclusion:**

Carers frequently described experiencing marginalisation, confidentiality being applied in exclusionary ways, as well as fragmented services, particularly around involuntary admission and discharge. Findings highlight modifiable targets for practice, including family-inclusive routines, transparent information-sharing and structured involvement in treatment and discharge planning, which are crucial given the elevated post-discharge suicide risk. Addressing these factors may reduce carer burden, strengthen trust and improve perceptions of quality and safety in inpatient psychiatric care. Limitations of this meta-review and directions for future research are discussed.

## Introduction

Informal carers are typically family members, friends or significant others who provide unpaid care and support to loved ones with physical, mental or emotional health needs (Colombo et al., 2011). As the number of primary informal carers for someone with mental illness in Australia is projected to grow by 18% by 2030 (Schofield et al., 2022), caregiver burden has become a significant challenge for family members and carers who assume primary responsibility for relatives with serious mental health conditions (Chan, 2011; Sealy, 2012). Caring for someone with a severe mental illness is often a long-term commitment, requiring ongoing crisis management and frequent engagement with emergency and hospital services (Miller et al., 2024). As a result, informal carers are typically the first to seek professional intervention for their loved one when they can no longer manage alone (Bradbury et al., 2017; Cairns et al., 2015). However, many carers report feeling dissatisfied with their interactions with these services, often describing them as negative or distressing experiences (Acres et al., 2019; Brennan et al., 2016; Dawson et al., 2012; Shankar and Muthuswamy, 2007). Thus, carers have advocated for hospital services to develop a better understanding of the lived experiences they face during periods of mental health crises for their loved ones (Askey et al., 2009; Gavois et al., 2006).

At times, individuals with severe mental illness may require inpatient hospitalisation. Advances in psychiatric treatment and a growing emphasis on human rights have led to the implementation of more evidence-based approaches within inpatient psychiatric settings, to the extent that mental health policies have increasingly mandated the inclusion of carers in treatment and care planning (Australian Government, 2010; Doody et al., 2017). However, while some studies have reported positive carer experiences within hospital environments (Karambelas et al., 2024), the broader literature highlights feelings of marginalisation or exclusion by mental health services for many carers (Cree et al., 2015; Ridley et al., 2010), despite ongoing policy attention to family-inclusive practice (Martin et al., 2017). For example, carers have expressed that their needs and views are frequently unrecognised and unaddressed (Foster et al., 2018; Stuart et al., 2020), which contributes to heightened levels of stress, burnout and other mental health difficulties (Abou Seif et al., 2022). Moreover, carers report that meaningful participation in their loved ones’ treatment remains limited in contexts involving involuntary inpatient admissions (Jankovic et al., 2011; Nurjannah et al., 2014; Petkari et al., 2021; Wilkinson and McAndrew, 2008).

The need to understand carers’ perceptions of inpatient psychiatric hospitals is further accentuated by the markedly high suicide rates following discharge from inpatient psychiatric facilities (Forte et al., 2019; Haglund et al., 2019; Madsen et al., 2020; Walsh et al., 2015). Post-discharge suicide rates have been reported to be as high as 44 times the global average, with the highest risk occurring within the first 3 months after discharge, during which care is typically transferred to families and informal carers (Chung et al., 2017). Given this context, failure to implement appropriate and effective discharge planning and continuity of care from inpatient psychiatric hospital to home can have severe repercussions (Clibbens et al., 2019). Therefore, clarifying carer’s perception of how inpatient hospitals are operated is essential for guiding the effective allocation of resources and encouraging policy changes that promote active carer involvement, with the aim of enhancing inpatient outcomes and ensuring continuity of care following discharge.

Families are increasingly being recognised as a vital source of support in the rehabilitation of individuals with severe mental illness, and there is a growing body of literature examining the roles and experiences of informal carers (Abou Seif et al., 2022; Albert and Simpson, 2015; Beardon et al., 2018; Lavoie, 2018; Rowe, 2012). Alongside this, more recent literature has increasingly emphasised relational recovery approaches, as well as the importance of recognising the wellbeing and recovery needs of carers and families (Mercuri and Petrakis, 2025; Wyder et al., 2022). The discourse surrounding informal caregiving has identified a range of important themes, including carer burden and unmet needs (Issac et al., 2022; Kuipers et al., 2006; Lloyd and Carson, 2005), challenges related to information-sharing and confidentiality (Gray et al., 2009; Slade et al., 2007), involvement in decision-making processes (Roulstone and Hudson, 2007; Stuart et al., 2020) and the quality of relationships between carers and mental health practitioners (Clarke, 2006; Krupnik et al., 2005; Wilkinson and McAndrew, 2008).

Despite this growing interest in the carer experience, there remains a need to consolidate and interpret the recurring factors that influence carers’ perceptions of inpatient psychiatric hospitals, particularly in comparison to the more extensively researched perspectives of inpatients and staff members (Modini et al., 2021; Thompson et al., 2024). Previous research has identified a range of factors influencing inpatients’ perceptions of psychiatric hospitals, including communication with staff, the ward environment, use of coercive measures, legal status, autonomy and expectations of care at both admission and discharge (Modini et al., 2021). Notably, staff perceptions were also found to align closely with those of inpatients across several of these domains (Thompson et al., 2024). Therefore, a comprehensive synthesis of the factors that influence carers’ perceptions of inpatient psychiatric hospitals is necessary to contextualise the findings within the broader body of work examining perceptions of inpatient psychiatric hospitals across key stakeholder groups.

Given the variety of systematic and other reviews that have previously examined specific subsets of factors influencing carers’ perceptions of inpatient psychiatric hospitals, a meta-review is the most appropriate and efficient methodological approach to synthesise this literature and address this research gap. A meta-review provides a comprehensive summary and synthesis of the best available evidence from previously published reviews on a topic of interest (Aromataris et al., 2020). The primary aim of this research is to systematically identify, summarise and synthesise evidence from existing systematic reviews and meta-analyses that explore the factors carers report as influencing their perceptions of inpatient psychiatric hospitals. In doing so, the findings are contextualised alongside recent meta-reviews of inpatients’ (Modini et al., 2021) and staff members’ (Thompson et al., 2024) perceptions to provide a more integrated understanding of the experiences and priorities of all groups involved in inpatient psychiatric care.

This review seeks to consolidate the existing literature, as well as highlight the persistence of these concerns despite increasing emphasis and advocacy surrounding family-inclusive and recovery-oriented mental health care. In addition, this review aims to contribute to ongoing discussion regarding best practice approaches to improve carers’ experiences of inpatient psychiatric hospitals, while also recognising what carers already perceive as beneficial.

## Method

### Protocol registration and reporting structure

The meta-review was conducted following the Preferred Reporting Items for Systematic Reviews and Meta-Analyses (PRISMA) guidelines (www.prisma-statement.org), and the protocol was registered on the PROSPERO international prospective register of systematic reviews in September 2024 (CRD42024552630).

### Search strategy

An initial scoping search on Google Scholar was conducted to map existing literature and identify key concepts relevant to carers’ perceptions of inpatient psychiatric hospitals. These findings informed the development of the eligibility criteria and search strategy. Searches were conducted using Medline, PsycINFO and Embase electronic databases (all via Ovid) for published systematic reviews and meta-analyses. Search terms combined keywords and subject headings relating to carers, inpatient psychiatric hospitals and perceptions of care, including “Caregiver,” “Carer,” “Psychiatric Hospital,” “Perception” and “Systematic Review.” The search was conducted in June 2024. Individual database search strategies are provided in Supplemental Appendix A.

### Eligibility criteria

Studies were included if they:

(a) were peer-reviewed systematic reviews, literature reviews or meta-analyses examining informal carers’ perceptions or experiences of inpatient psychiatric hospitals;
(b) assessed carer perceptions relating to patients who spent at least one night in an inpatient setting;
(c) used a broad definition of caregivers, including family members, friends or significant others involved in supporting the individual during the admission;
(d) included adult, adolescent, forensic, geriatric or emergency psychiatric inpatient wards;
(e) were published in English, with no year restrictions;
(f) synthesised primary research using qualitative, quantitative or mixed-methods designs.

Studies were excluded if they:

(a) focused solely on inpatient or staff perceptions;
(b) reviewed psychometric properties of quality measures;
(c) presented only author opinions or commentaries;
(d) focused on paid carers, non-carers or carers of individuals with physical conditions unless comorbid with a severe mental illness.

Where reviews included multiple stakeholders, only carer-specific findings were extracted and synthesised. Similarly, when reviews included both inpatient and community mental health settings, only findings specifically relating to inpatient psychiatric care were included.

### Selection process

Search results were imported into EndNote for duplicate removal. Two researchers (KK and MM) independently screened titles and abstracts according to the eligibility criteria. Full-text review was subsequently undertaken independently by the same researchers to determine final inclusion. Disagreements at any stage were resolved through discussion with a third researcher (MJA) until consensus was achieved.

### Data extraction

The first author (KK) conducted the data extraction. Extracted information included citation details, review type, quality rating, relevant aims, key findings and reported themes relevant to carers’ perceptions of inpatient psychiatric hospitals (Table 1). Reviews incorporating qualitative, quantitative and mixed-methods findings were included. Quantitative findings were extracted where they contributed to understanding carers’ perceptions or experiences and were synthesised alongside qualitative findings during thematic synthesis.

**Table 1.** Included studies.

Author/s and year of publication	Type of review	Number of included studies	Quality rating	Relevant aim/s^a^	Key finding/s^b^	Theme/s extracted from this review^c^
Abou Seif et al. (2022)	Systematic review: included only qualitative studies.	12	Moderate	Review research on informal carers’ experiences of adult inpatient mental health care to identify key themes, unmet needs and inform improvements in carer inclusion, support and policy.	Carers reported experiencing significant emotional distress, feeling excluded from care decisions and dissatisfaction with care quality, highlighting the need for greater support, inclusion and partnership. Four key themes were identified, including carers’ emotional turmoil, invisibility and exclusion from care, concerns about care quality and strained relationships with staff and loved ones.	Exclusion and disempowerment; communication and information deficits; emotional and care burden; systemic barriers and crisis-driven pathways; desire for partnership and collaborative care.
Alyafei et al. (2021)	Systematic review: Included qualitative and mixed-methods studies.	12	Moderate	Synthesise caregivers’ experiences of severe mental illness in the Middle East to inform culturally relevant support.	Family caregivers in the Middle East face substantial emotional, physical and financial burdens, compounded by stigma, strained relationships and limited support. These challenges and a lack of illness knowledge significantly impact their mental health, despite reliance on religious coping strategies.	Communication and information deficits; systemic barriers and crisis-driven pathways.
Bartl et al. (2024)	Systematic review: Included only qualitative studies.	24	Moderate	Synthesis of qualitative evidence on carers’ experiences of assessment and involuntary hospital admissions under mental health legislation to inform policy and service improvements.	Compulsory mental health admissions are often experienced as negative, traumatic and disempowering; however, some see them as helpful. Carers described emotional strain, exclusion from decision-making and a lack of support, highlighting the need for more collaborative and respectful care approaches.	Exclusion and disempowerment; communication and information deficits; emotional and care burden; systemic barriers and crisis-driven pathways; desire for partnership and collaborative care.
Clibbens et al. (2019)	Integrative review: Included qualitative, quantitative and mixed-methods studies.	11	Low	Investigate and synthesise what is known about the experiences of caregivers of people with serious mental health problems during transitions between adult mental health services.	Caregivers experience heightened stress during service transitions, facing insufficient information, limited involvement in care decisions and challenges accessing services. They often feel unsupported, take on significant responsibilities and struggle with emotional and practical burdens.	Exclusion and disempowerment; communication and information deficits; emotional and care burden; systemic barriers and crisis-driven pathways; desire for partnership and collaborative care.
Dawson et al. (2012)	Systematic review: Included qualitative and mixed-methods studies.	22	Low	Synthesise qualitative evidence on carers’ experiences of supporting a relative with first-episode psychosis, focusing especially on their experiences before and during help-seeking.	Carers of relatives with first-episode psychosis often experienced delays in recognising illness and seeking help, influenced by stigma and lack of knowledge. They faced barriers from reluctant relatives and unhelpful social networks, with crises or severe symptoms usually prompting eventual help-seeking.	Communication and information deficits; emotional and care burden; systemic barriers and crisis-driven pathways.
Doody et al. (2017)	Integrative review: Included qualitative, quantitative and mixed-methods studies.	15	Low	Explore families’ experiences of involvement in care planning within adult mental health services.	Families felt largely excluded from care planning in adult mental health services, experiencing powerlessness, lack of communication and negative attitudes from professionals. Confidentiality was often used as a barrier, and families perceived a lack of genuine partnership and appreciation of their role, highlighting the need for more collaborative approaches.	Exclusion and disempowerment; communication and information deficits; desire for partnership and collaborative care.
Ho et al. (2022)	Integrative review: Included only qualitative studies.	7	Moderate	Explore carers’ experiences and views regarding the physical health care of people with severe mental illness, by identifying perceived facilitators, barriers and their roles in supporting physical health.	Carers play crucial roles in supporting the physical health of people with severe mental illness; however, they face major barriers such as poor service coordination, lack of communication, stigma, diagnostic overshadowing and feeling excluded by health professionals. Carers highlight the need for better support, clear information and stronger involvement in healthcare planning.	Exclusion and disempowerment; communication and information deficits; emotional and care burden; desire for partnership and collaborative care.
Issac et al. (2022)	Systematic review & meta-synthesis: Included only qualitative studies.	38	Moderate	Identify and synthesise qualitative findings on the needs, challenges and coping strategies of primary caregivers of family members diagnosed with schizophrenia.	Primary caregivers of people with schizophrenia face significant challenges including financial strain, violence, treatment non-compliance and social stigma, leading to emotional and physical burdens. They express needs for better information, support services and community resources. Caregivers adopt various coping strategies, such as problem-focused, emotional, behavioural, social and religious coping.	Communication and information deficits; emotional and care burden; systemic barriers and crisis-driven pathways; desire for partnership and collaborative care.
Joung and Shin (2023)	Integrative review: Included qualitative, quantitative and mixed-methods studies.	9	Low	Explore and synthesise the challenges experienced by family caregivers of individuals with severe mental illness during the COVID-19 pandemic, to inform mental health policies and future interventions.	Family caregivers experienced increased care burden, significant physical and mental health problems (such as fatigue, anxiety and depression) and major difficulties accessing healthcare services during the COVID-19 pandemic. These challenges highlighted the urgent need for better support systems and tailored interventions for caregivers in crisis situations.	Systemic barriers and crisis-driven pathways.
Lascelles et al. (2024)	Systematic review: Included qualitative, quantitative and mixed-methods studies.	31	Low	Understand the experiences and needs of adults who care for another adult at risk of suicide.	Adult informal carers of people at risk of suicide experience profound mental, physical and social burdens, including constant fear, uncertainty and hypervigilance. Their relationships often change and become strained, and they feel excluded and unsupported by healthcare services. Carers want to be better informed, involved and holistically supported to address their emotional and practical needs.	Exclusion and disempowerment; communication and information deficits; emotional and care burden; systemic barriers and crisis-driven pathways; desire for partnership and collaborative care.
MacDonald et al. (2021)	Meta-synthesis review: Included only qualitative studies.	31	Moderate	Understand the experiences of young people and their carers when navigating pathways to mental health services, to inform service improvements and support reform efforts grounded in their perspectives.	Young people and their carers experienced complex, fragmented and often delayed pathways to mental health care, marked by structural barriers (like cost, transport and eligibility), low mental health literacy, stigma and poor service coordination. Positive experiences included accessible, flexible and compassionate care, while negative experiences involved feeling misunderstood, excluded or disrespected.	Exclusion and disempowerment; communication and information deficits; emotional and care burden; systemic barriers and crisis-driven pathways; desire for partnership and collaborative care.
Moyo et al. (2022)	Systematic review: Included qualitative and mixed-methods studies.	49	Low	Update and extend previous work by examining international carer views to inform policy and practice globally.	Carers felt excluded from care, reported their concerns were not taken seriously and wanted greater involvement and respect. The findings emphasise the need for mental health nurses to adopt more collaborative, recovery-focused and inclusive approaches.	Exclusion and disempowerment; communication and information deficits; desire for partnership and collaborative care.
Reupert et al. (2023)	Systematic review: Included only qualitative studies.	8	Moderate	Synthesise qualitative research on the experiences of parents, children and other family members when a parent with dependent children is hospitalised for their mental illness, to inform policy and improve family-focused mental health care.	Family members experience significant pressure and increased responsibilities when a parent is hospitalised for mental illness, including challenges in managing visits and caring for children. Children often face emotional distress, lack information and need guidance and support. Overall, families feel their needs are overlooked, highlighting the need for better communication, psychoeducation and family-focused support in mental health services.	Exclusion and disempowerment; communication and information deficits; emotional and care burden; systemic barriers and crisis-driven pathways.
Stuart et al. (2020)	Systematic review & meta-synthesis: Included only qualitative studies.	23	Low	Synthesise evidence on carers’ experiences of the assessment and detention of family members or friends under mental health legislation, to inform policy and practice improvements.	Carers described the detention of their relatives as emotionally distressing and traumatic, marked by feelings of being excluded, undervalued and burdened by increased responsibilities. They reported inadequate support, lack of involvement in decision-making and poor communication from professionals.	Exclusion and disempowerment; communication and information deficits; emotional and care burden; systemic barriers and crisis-driven pathways; desire for partnership and collaborative care.
Sugiura et al. (2020)	Systematic review & meta-synthesis: Included only qualitative studies.	37	Low	Describe the experiences of informal carers during decision-making for involuntary psychiatric admission and throughout the subsequent admission and to explore what supports service users need to have their rights and preferences respected.	Informal carers felt responsible for initiating involuntary admissions but also experienced guilt, fear, anger and worry about harming their relationships with the person admitted. They found the admission process confusing, felt unsupported by mental health services and struggled to get clear information and guidance. Carers often felt excluded from treatment planning, which strained family bonds and increased their emotional burden.	Exclusion and disempowerment; communication and information deficits; emotional and care burden; systemic barriers and crisis-driven pathways; desire for partnership and collaborative care.
Vestphal et al. (2023)	Evidence synthesis: Included only qualitative studies.	13	Moderate	Investigate and synthesise existing research on family caregivers’ experiences in forensic mental health care, particularly focusing on their experiences with facilitators and barriers to involvement in care and treatment.	Family caregivers in forensic mental health care experienced ongoing violence, heavy responsibility and difficult collaboration with professionals. They felt excluded from care, struggled with guilt and took on intense, persistent roles to protect and advocate for their relatives. These challenges led to feelings of powerlessness and emotional distress.	Exclusion and disempowerment; communication and information deficits; emotional and care burden; systemic barriers and crisis-driven pathways; desire for partnership and collaborative care.
Zhao et al. (2023)	Meta-aggregative synthesis: Included qualitative and mixed-methods studies.	24	Moderate	Systematically review and synthesise qualitative research on the experiences, preferences and expectations of parents caring for young people who self-harm, to inform clinical practice and support family-based interventions.	Parents of young people who self-harm often felt unsupported and excluded by mental health services and lacked knowledge and resources to help their child effectively. Parents expressed a strong need for psychoeducation, emotional support and greater involvement in care.	Exclusion and disempowerment; communication and information deficits; emotional and care burden; systemic barriers and crisis-driven pathways; desire for partnership and collaborative care.

a Only relevant aims that pertain to carer perception of a psychiatric hospital are reported.

b Only relevant results that correspond with the aims of this review are reported.

c Extracted theme(s) = Theme(s) identified via thematic synthesis of the reviews included in this meta-review.

### Quality assessment of included reviews

Methodological quality was assessed independently by two researchers (KK and MM) using an adapted version of the AMSTAR-2 appraisal tool (Shea et al., 2017). Adaptations were informed by the Joanna Briggs Institute (JBI, 2017) critical appraisal guidance to better accommodate qualitative and mixed-methods reviews, consistent with approaches taken in previous meta-reviews (Modini et al., 2021; Thompson et al., 2024).

Several AMSTAR-2 items were modified to reflect the qualitative focus of many included reviews. For example, references to intervention-based criteria were replaced with criteria relevant to assessing whether included qualitative non-intervention studies were described in adequate detail, including population, phenomenon of interest and context (PICo; Stern et al., 2014). In addition, any references to randomised controlled trials (RCTs) and non-randomised studies of interventions (NRSIs) in the AMSTAR-2 were disregarded and replaced with considerations for qualitative research, such as explaining why specific studies were selected, rather than justifying the inclusion of RCTs or NRSIs. Consideration was also given to publication year when assessing reporting quality and risk of bias, as some included studies predated the updated PRISMA 2020 Checklist (Page et al., 2021) and instead adhered to the PRISMA 2009 Checklist (Moher et al., 2010). Finally, to accommodate qualitative studies, item 14 was modified to “Did the review authors provide a satisfactory explanation and discussion of limitations, including their impact on the results, conclusions and any recommendations?”

AMSTAR-2 recommends against creating an overall score, as some critical domains are deemed more important than others, suggesting providing overall confidence ratings instead (Shea et al., 2017). Confidence ratings are as follows: High (none or one non-critical weakness), Moderate (more than one non-critical weakness), Low (one critical flaw with or without non-critical weaknesses) and Critically Low (more than one critical flaw with or without non-critical weaknesses). Reviews deemed “Critically Low” were excluded from the final review to increase confidence in the findings. Any disagreements about a study’s quality rating were referred to a third researcher (MJA) for resolution.

### Data synthesis

Given the predominance of qualitative findings in the included studies, a thematic synthesis approach was undertaken, guided by Thomas and Harden’s (2008) methods for synthesising qualitative research. This meta-review therefore represents a qualitative thematic synthesis rather than a statistical meta-analysis due to the lack of sufficient quantitative data.

This method involved four stages to descriptively synthesise and integrate the findings from the included reviews and then analyse the factors influencing carers’ perceptions of inpatient psychiatric hospitals. The initial phase involved familiarisation with the data through repeated reading of each review to ensure comprehensive understanding of the content (Green et al., 2007).

The second phase involved a systematic coding process of examining and organising the information within each dataset. A coding frame was developed, consisting of codes derived through a line-by-line examination of the synthesised data presented in the included reviews (Thomas and Harden, 2008). Data relating to carers’ experiences and perceptions of inpatient psychiatric care were coded using this framework by the first researcher (KK). Quantitative findings were included where they contributed to understanding these experiences.

The third phase involved grouping and categorising the codes into broader descriptive themes. These themes reflected patterns and commonalities across studies despite variations in methodology and study design (Thomas and Harden, 2008). This step enabled the synthesis of similar experiences identified in one review compared with those from others, facilitating the pooling of data to produce a more comprehensive summary across different settings (Modini et al., 2021).

The final phase of the synthesis involved generating analytical themes. This stage was informed by the interpretative judgement and insights of the reviewers, as described by Thomas and Harden (2008). Themes were synthesised across the included reviews and refined in relation to the overall aims of the meta-review. Identifying themes, rather than categories, strengthens the evidence by providing a more detailed explanation of the social phenomena observed in the review (Green et al., 2007; Lachal et al., 2017). Thematic extraction was considered complete once data saturation was achieved, which was defined as the point at which no new themes emerged from the data (Fusch and Ness, 2015; Saunders et al., 2018).

To ensure all extracted themes generated could be inferred from the included studies, a second researcher (MM) independently conducted a reliability analysis. There was no disagreement between researchers around the themes generated, as such a full consensus was reached regarding the final set of extracted themes.

## Results

Through database searching, 8247 articles were identified after the removal of duplicates. Out of the 58 studies selected for full-text screening, a further 31 were excluded due to not meeting the eligibility criteria. Following the quality assessment using the AMSTAR-2 (Shea et al., 2017), 10 were excluded due to being deemed “Critically Low” in methodological quality. This resulted in a total of 17 studies included in the final meta-review (Abou Seif et al., 2022; Alyafei et al., 2021; Bartl et al., 2024; Clibbens et al., 2019; Dawson et al., 2012; Doody et al., 2017; Ho et al., 2022; Issac et al., 2022; Joung and Shin, 2023; Lascelles et al., 2024; MacDonald et al., 2021; Moyo et al., 2022; Reupert et al., 2023; Stuart et al., 2020; Sugiura et al., 2020; Vestphal et al., 2023; Zhao et al., 2023). The search process is outlined in Figure 1.

**Figure 1.**
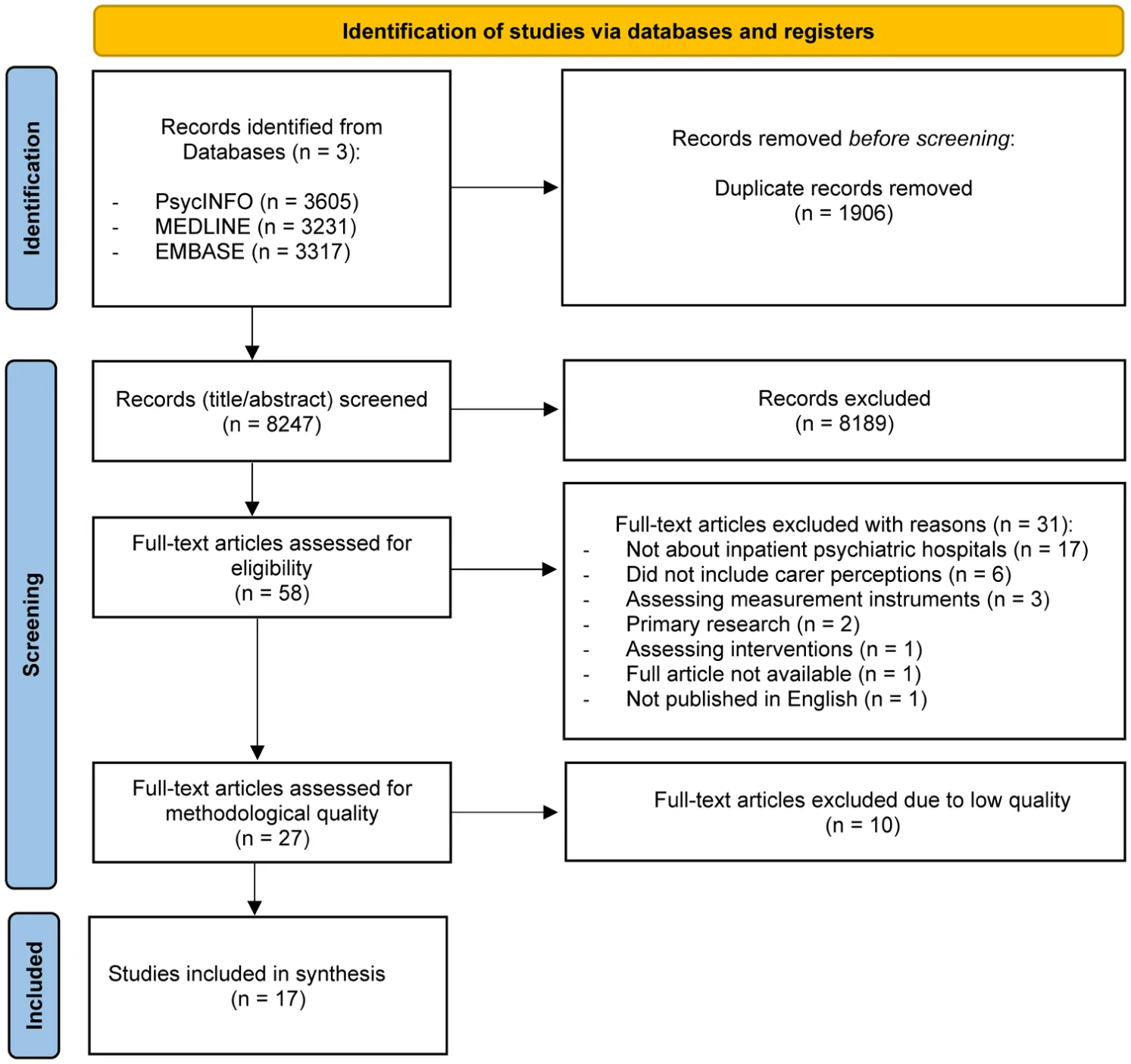
PRISMA flow diagram.

Thematic synthesis identified five overarching and interconnected themes that influenced carer perceptions of psychiatric hospitals: exclusion and disempowerment, communication and information deficits, emotional and care burden, systemic barriers and crisis-driven pathways and desire for partnership and collaborative care. These themes were developed through an iterative process of grouping recurrent findings and patterns reported across the included reviews, with representative findings linked to each analytical theme presented in Table 1.

### Exclusion and disempowerment

Thirteen of the included reviews revealed a dominant theme of the exclusion and disempowerment of carers within inpatient psychiatric settings. Carers consistently described feeling invisible, marginalised and excluded from decisions that directly affected both themselves and their loved ones (Abou Seif et al., 2022; Bartl et al., 2024; Clibbens et al., 2019; Doody et al., 2017; MacDonald et al., 2021; Reupert et al., 2023; Zhao et al., 2023). Despite their experiential knowledge of the inpatient’s needs, they were often reduced to the role of informants and were expected to supply background information while rarely being acknowledged as legitimate partners in treatment planning or clinical decision-making (Doody et al., 2017; Lascelles et al., 2024; Stuart et al., 2020; Sugiura et al., 2020; Vestphal et al., 2023).

This lack of recognition was compounded by carers’ reports of being dismissed, blamed, judged or treated as obstructive, which undermined their sense of legitimacy (Abou Seif et al., 2022; Bartl et al., 2024; Clibbens et al., 2019; Doody et al., 2017; MacDonald et al., 2021; Stuart et al., 2020). Such experiences denied carers the opportunity to engage as equal partners and devalued their insights into loved ones’ triggers and behaviours, which sometimes lead to preventable escalations that could have been averted with greater collaboration (Bartl et al., 2024; Moyo et al., 2022; Stuart et al., 2020; Sugiura et al., 2020; Vestphal et al., 2023). The failure to acknowledge carers’ knowledge deepened feelings of disempowerment and undermined confidence in staff competence and compassion (Ho et al., 2022; Zhao et al., 2023). Over time, these dynamics created an “us versus them” divide between families and professionals, eroding trust and making collaborative partnership increasingly difficult (Abou Seif et al., 2022; Doody et al., 2017; Vestphal et al., 2023).

These cumulative experiences influenced carers’ views of inpatient psychiatric hospitals as institutions that disregard their expertise and position them as outsiders in the care process. The overarching theme of exclusion and disempowerment was further intertwined with other recurring themes including communication and information deficits, emotional and care burden, systemic barriers and crisis-driven pathways and carers’ desire for partnership, which heightened carers’ overall experience of marginalisation in inpatient settings.

### Communication and information deficits

Another prevalent theme identified across 16 of the included reviews was the lack of effective communication and information-sharing, which carers consistently described as a major source of stress, confusion and dissatisfaction with inpatient psychiatric hospitals. Reviews reported carer experiences of inadequate, unclear or poorly timed information about diagnoses, treatment plans, discharge arrangements and relapse prevention (Abou Seif et al., 2022; Alyafei et al., 2021; Clibbens et al., 2019; Issac et al., 2022; MacDonald et al., 2021; Moyo et al., 2022; Stuart et al., 2020). This lack of clarity often left carers feeling uninformed and unprepared to support recovery at home, particularly at critical transition points such as discharge. Carers reported uncertainty about how to manage medication, recognise relapse warning signs and navigate appropriate help-seeking pathways (Bartl et al., 2024; Clibbens et al., 2019; Lascelles et al., 2024; Stuart et al., 2020; Sugiura et al., 2020; Zhao et al., 2023).

Carers also highlighted a strong unmet need for education and support. They frequently described being given little or no guidance about mental illness, treatment options or strategies for managing illness-related behaviours despite being expected to take on the caregiving responsibilities after discharge (Alyafei et al., 2021; Bartl et al., 2024; Clibbens et al., 2019; Ho et al., 2022; Issac et al., 2022; Lascelles et al., 2024; Reupert et al., 2023; Sugiura et al., 2020). Where staff provided psychoeducation, accessible explanations or empathetic engagement, carers reported relief, greater confidence and improved trust in services (Lascelles et al., 2024; MacDonald et al., 2021). However, such experiences were the exception rather than the norm.

Confidentiality was often cited as a further barrier to collaboration, with carers acknowledging the importance of privacy but experiencing its application as exclusionary (Bartl et al., 2024; Clibbens et al., 2019; Dawson et al., 2012; Lascelles et al., 2024; Stuart et al., 2020; Sugiura et al., 2020). Families described how confidentiality was routinely used to justify withholding essential information, leaving them “in the dark” about medication changes, transfers or treatment progress (Clibbens et al., 2019; Doody et al., 2017; Moyo et al., 2022; Reupert et al., 2023; Sugiura et al., 2020; Vestphal et al., 2023). In many cases, critical updates were provided only at the last minute or not at all, which created a sense of uncertainty for carers responsible for ongoing care at home (Abou Seif et al., 2022; MacDonald et al., 2021). These one-sided communication practices were particularly frustrating for carers, who were often relied upon to supply contextual knowledge about their loved one but rarely received reciprocal information in return.

Poor communication was further demonstrated by reports of inconsistent and dismissive staff practices, including vague updates, cold or insensitive delivery of information and conflicting messages between professionals (Clibbens et al., 2019; Dawson et al., 2012; Doody et al., 2017; Ho et al., 2022; MacDonald et al., 2021; Stuart et al., 2020; Sugiura et al., 2020). Such experiences not only undermined carers’ confidence in the quality of hospital care but also reinforced their sense of being disregarded, unsupported and excluded from meaningful partnership with staff.

### Emotional and care burden

The emotional and care burden experienced by carers during a loved one’s psychiatric hospitalisation was identified across 13 reviews. Carers described hospitalisation as eliciting conflicting emotions. While admission often brought relief and temporary respite (Issac et al., 2022; MacDonald et al., 2021; Stuart et al., 2020; Vestphal et al., 2023), it also generated feelings of guilt, fear and trauma, particularly when carers had played a role in initiating care (Abou Seif et al., 2022; Bartl et al., 2024; Clibbens et al., 2019; Dawson et al., 2012; Sugiura et al., 2020). Many reported significant psychological strain and emotional exhaustion, exacerbated during crises and especially in the context of coercive or involuntary admissions (Clibbens et al., 2019; Stuart et al., 2020; Sugiura et al., 2020). These events were often described as profoundly disorienting and distressing, leaving carers powerless and unsupported in navigating the process.

The burden extended beyond emotional strain to practical and clinical responsibilities. Carers frequently assumed additional roles, such as advocating for treatment, managing medications or bridging gaps in communication, often as a direct result of systemic failures (Bartl et al., 2024; Clibbens et al., 2019; Dawson et al., 2012; Ho et al., 2022; Reupert et al., 2023; Stuart et al., 2020; Vestphal et al., 2023). These additional responsibilities compounded their stress and contributed to a sense of role overload.

In addition, carers felt that their own emotional needs and distress went unnoticed by professionals, leaving them isolated, undervalued and unsupported, despite the expectation that they would assume primary responsibility once the person was discharged (Bartl et al., 2024; Lascelles et al., 2024; Zhao et al., 2023). The combination of high emotional intensity, role overload and insufficient recognition highlights the extent to which hospitalisation of their loved one was experienced as overwhelming and distressing for carers.

### Systemic barriers and crisis-driven pathways

The impact of systemic barriers and crisis-driven pathways on carers’ experiences of inpatient psychiatric services was identified in 14 reviews. Carers frequently described services as under-resourced, fragmented and reactive, with access to care delayed until the crisis point (Abou Seif et al., 2022; Clibbens et al., 2019; Dawson et al., 2012; Joung and Shin, 2023; Lascelles et al., 2024; MacDonald et al., 2021; Stuart et al., 2020; Sugiura et al., 2020; Vestphal et al., 2023). Carers reported struggling to navigate complex and confusing pathways, encountering premature discharges, inadequate discharge planning and poor coordination between services (Abou Seif et al., 2022; Bartl et al., 2024; Clibbens et al., 2019; Issac et al., 2022; Lascelles et al., 2024; MacDonald et al., 2021; Reupert et al., 2023; Stuart et al., 2020; Sugiura et al.,2020; Vestphal et al., 2023). These systemic barriers left carers having to manage escalating risk on their own and diminished trust in the system’s capacity to provide effective and timely care.

Concerns about the quality and humanity of inpatient care were also recurrent. Carers perceived an over-reliance on medication, limited therapeutic engagement and a custodial rather than recovery-oriented model of care (Bartl et al., 2024; Stuart et al., 2020). These accounts reinforced perceptions of hospitals as dismissive, unsafe or even dehumanising, leading to institutional mistrust (Abou Seif et al., 2022; Bartl et al., 2024; Issac et al., 2022; Lascelles et al., 2024; Reupert et al., 2023; Stuart et al., 2020). These issues were particularly prominent during involuntary admissions, which carers often described as traumatic, disorienting and isolating (Bartl et al., 2024; Clibbens et al., 2019; Dawson et al., 2012; MacDonald et al., 2021; Stuart et al., 2020). The exclusion from assessment and planning, combined with dismissive staff attitudes and minimal guidance left many carers feeling unprepared for discharge, receiving little more than a “bag of medication” (Bartl et al., 2024).

Some reviews highlighted the impact of structural inequities including financial costs, geographic distance and pandemic-related restrictions, which limited opportunities for engagement and magnified the sense of isolation and vulnerability for carers who already felt marginalised within the system (Alyafei et al., 2021; Dawson et al., 2012; Joung and Shin, 2023; Zhao et al., 2023). These barriers reinforced carers’ mistrust of inpatient services and contributed to enduring perceptions of hospitals as institutions that neglect both inpatient recovery and carer needs.

### Desire for partnership and collaborative care

Across 13 of the included reviews, carers consistently expressed a strong desire to be acknowledged as partners in care (Abou Seif et al., 2022; Bartl et al., 2024; Clibbens et al., 2019; Ho et al., 2022; Lascelles et al., 2024; Vestphal et al., 2023; Zhao et al., 2023). They emphasised the importance of being recognised for their lived experience and valued when professionals engaged them with transparency, empathy and respect (Bartl et al., 2024; Doody et al., 2017; Lascelles et al., 2024). Carers highlighted that their insights into a loved one’s history, behaviours and triggers could be invaluable for care planning and safety, yet these contributions were often overlooked.

The evidence demonstrated that when carers were meaningfully included through timely communication, acknowledgement of expertise and involvement in treatment discussions or discharge planning, their experiences of hospitalisation were significantly more positive (Abou Seif et al., 2022; Bartl et al., 2024; Clibbens et al., 2019; Issac et al., 2022; MacDonald et al., 2021). Carers that felt included reported feelings of relief, empowerment and confidence in their ability to support recovery after discharge, as well as improved trust in staff and services (Abou Seif et al., 2022; Clibbens et al., 2019; Ho et al., 2022; Issac et al., 2022; Lascelles et al., 2024; Moyo et al., 2022; Stuart et al., 2020; Sugiura et al., 2020). It was found that partnership approaches not only enhanced carers’ wellbeing but also improved perceptions of service quality and safety, reinforcing the idea that collaborative, family-inclusive practices are essential to recovery-oriented care.

## Discussion

This meta-review aimed to identify, summarise and synthesise the evidence examining carers’ perceptions of psychiatric hospitals. Across 17 included studies, five themes that consistently shaped carers’ perceptions emerged: exclusion and disempowerment, communication and information deficits, emotional and care burden, systemic barriers and crisis-driven pathways and the desire for partnership and collaborative care. Together, these themes highlight that carers’ dissatisfaction with psychiatric hospitals stems from overlapping issues shaped by broader systemic and organisational dynamics within inpatient mental health care.

When viewed alongside existing syntheses of inpatient and staff perceptions, the findings of this meta-review reveal important differences in how psychiatric hospitals are experienced. Carers’ perceptions were influenced by experiences of exclusion, lack of support, lack of recognition of their expertise and the displacement of responsibility for ongoing care after discharge. In contrast, inpatients’ perceptions centred on ward relationships, autonomy, the ward environment and experiences of coercion, with therapeutic rapport and respect emerging as the most consistently valued aspects of hospital care (Modini et al., 2021). On the other hand, staff perspectives focused on safety, service delivery pressures and the challenges of balancing therapeutic engagement with resource constraints and risk management (Thompson et al., 2024). While all three groups identified trust, safety and the quality of partnerships as critical, their experiential perspectives differed significantly, particularly where inpatients prioritise autonomy and privacy, while carers seek greater involvement in treatment and discharge planning. This underscores the need for integrated approaches that acknowledge and bridge these perspectives, ensuring that carer, inpatient and staff experiences are not treated in isolation.

In addition, the findings of this meta-review revealed unsurprising unique barriers faced by carers. Unlike inpatients or staff, carers are largely removed from the day-to-day routines and interactions that shape experiences within the ward environment. Consequently, their perceptions appear to be less influenced by the immediate operational aspects of hospital life and more by overarching concerns, including communication, involvement in decision-making and the transfer of responsibility for care following discharge. As such, the theme of exclusion and disempowerment was particularly pronounced, with many carers feeling invisible within the system, dismissed by professionals or included only as passive informants, rather than active partners in decision-making. This exclusion was compounded by communication deficits, with carers reporting that confidentiality was often applied in ways that left them uninformed about treatment and discharge planning. While confidentiality and privacy legislation are critical to protecting inpatient rights, these practices were described not only as frustrating but also as actively undermining carers’ capacity to provide safe and effective support at home. Staff are therefore challenged to navigate complex ethical and legal responsibilities outlined in the relevant policy and legislation to their specific health district/country, while continually balancing inpatient autonomy, confidentiality obligations and meaningful carer involvement.

Carers also frequently articulated their experiences in ways that consistently reflected an overarching sense of dissatisfaction and a need for their perspectives to be acknowledged. For example, the findings convey carers’ broader frustrations with assuming additional responsibilities, such as advocating for treatment, bridging communication gaps and providing emotional support, while their own needs went unrecognised. This is notable given the increasing emphasis within contemporary recovery-oriented literature on recognising carers and families as individuals with their own support and recovery needs (Mercuri and Petrakis, 2025; Wyder et al., 2022). Psychiatric hospitals were often described as under-resourced, fragmented and reactive, leading to premature discharges, poor coordination between services and an over-reliance on medication, which reinforced carers’ mistrust of hospitals and deepened their perceptions of psychiatric wards as custodial rather than therapeutic. The consequences of these practices are particularly concerning given the elevated risk of suicide following discharge from psychiatric hospitals (Chung et al., 2017). Therefore, inadequate discharge planning and poor continuity of care represent more than organisational shortcomings as they pose significant risks with potentially severe consequences.

Importantly, when carers were offered clear, timely information, acknowledged for their expertise and involved in planning, their perceptions shifted markedly. Such experiences were described as empowering, improving trust in services and instilling confidence in carers’ ability to support recovery after discharge. These positive experiences have also been associated with significant improvements in patient symptom severity and quality of life (Fleury et al., 2008; Norman et al., 2005; Schofield et al., 2001; Tempier et al., 2013), which highlights the critical role of carer involvement not only in enhancing perceptions of care but also in safeguarding patient outcomes during this high-risk period. This suggests that collaborative care does not necessarily undermine inpatient autonomy, but may strengthen continuity, trust and shared understanding when implemented appropriately.

Encouragingly, the evidence suggests that carers’ negative experiences are not inevitable but modifiable. Carers consistently expressed a strong desire for partnership and collaborative care, with meaningful inclusion leading to positive outcomes for both carers and inpatients. These findings align with international policy and practice frameworks that emphasise family inclusion as integral to recovery-oriented care (Baker et al., 2019; Department of Health and Social Care, 2024; Misra et al., 2025). However, despite the translation of these frameworks into practice being well supported by evidence of reduced relapse rates, carer distress and reliance on hospital services (Cameron et al., 2024), the implementation remains inconsistent and undermined by systemic and organisational barriers such as confidentiality, professional attitudes and workload pressures (Thorburn, 2019). Notably, many of the concerns identified across the included studies appear consistent across both older and contemporary literature despite substantial policy and service reform efforts. This points to the need for further improvement and reform, whereby embedding transparent communication, greater reciprocal information-sharing and genuine partnership into inpatient practice may help to rebuild trust, ease carer burden and promote a more collaborative model of care.

### Strengths and limitations

The strengths of this meta-review include its comprehensive and systematic search strategy across a broad area and the use of AMSTAR-2 (Shea et al., 2017), a validated tool for appraising methodological quality. The exclusion of reviews rated as “Critically Low” enhanced the reliability of findings by ensuring that only higher-quality evidence contributed to the synthesis. The thematic synthesis allowed for the integration of diverse perspectives across multiple reviews, strengthening confidence in the selection of the identified themes.

Nevertheless, several limitations must be acknowledged. First, a meta-review is constrained by the limitations of the primary studies included within systematic reviews. Many primary studies relied on retrospective accounts, raising the possibility of recall bias, and often sampled carers who were more motivated to share negative experiences. In addition, self-selection bias in the original studies cannot be ruled out. Carers who had experienced significant dissatisfaction or challenges with mental health services may have been more inclined to participate in research, which could partly account for the more negatively oriented findings observed in this review compared with those in inpatient and staff-focused reviews. Second, some included reviews did not clearly distinguish whether carers’ perceptions related specifically to inpatient or outpatient settings. To maintain the focus of this study, data that could not be confidently attributed to inpatient contexts were excluded, which may have led to omission of relevant experiences. Finally, thematic synthesis involves an interpretive process that introduces a degree of interpretive bias and subjectivity; however, steps were taken to mitigate this through independent reviewing and cross-checking.

For clinical practice, these findings point towards the need for hospitals to move beyond rhetorical commitments to family involvement and actively implement collaborative, family-inclusive approaches as routine practice (Martin et al., 2017). This requires system-level reforms that dismantle exclusionary practices and address barriers to partnership by prioritising transparent information-sharing, active carer participation in treatment and discharge planning and recognition of carers’ expertise as central to recovery-oriented care (Cameron et al., 2024). While patient confidentiality and privacy must be respected, failure to provide timely and essential information to carer’s must be balanced with the need to address the markedly high inpatient suicide rate post discharge.

Organisational change should establish structures that also attend to carers’ emotional and practical needs given the burden placed on them for post-discharge care, which should be supported by staff training on ways to incorporate this into overall patient care. By implementing these measures, hospitals can improve carers’ experiences and reduce reliance on crisis-driven pathways, which would directly enhance patient outcomes, continuity of care and institutional trust.

Future research should evaluate the impact of family-inclusive interventions on both carer and patient outcomes, develop tools to measure carer experiences in a consistent way and explore cultural and contextual differences in how carers engage with psychiatric services. By centring carers’ voices and embedding collaborative practice, psychiatric hospitals can move closer to creating therapeutic, recovery-oriented environments that support not only patients but also the families and friends who play a critical role in their ongoing care.

## Conclusions

This meta-review demonstrates that carers’ perceptions of psychiatric hospitals are strongly shaped by exclusion, poor communication, emotional burden, systemic inadequacies and the absence of meaningful partnership. These findings highlight not only the need to address and recognise the systemic barriers that marginalise carers and limit their involvement in care but also the importance of translating existing knowledge into meaningful clinical and systemic change. Encouragingly, these findings also demonstrate that inclusion, respect and collaboration are both feasible and potentially transformative, with the capacity to improve carer wellbeing, strengthen trust and enhance perceptions of service quality and safety.

## Supplemental Material

sj-docx-1-anp-10.1177_00048674261478446 – Supplemental material for Carer perceptions of inpatient psychiatric hospitals and the treatment of their loved ones: A meta-review of the literature Supplemental material, sj-docx-1-anp-10.1177_00048674261478446 for Carer perceptions of inpatient psychiatric hospitals and the treatment of their loved ones: A meta-review of the literature by Katarina Khitra, Matthew Modini and Maree J Abbott in Australian & New Zealand Journal of Psychiatry
